# Brief but not bland

**DOI:** 10.1038/s44319-025-00570-x

**Published:** 2025-09-08

**Authors:** Howy Jacobs

**Affiliations:** Faculty of Medicine and Health Technology, Tampere University, Tampere, Finland

**Keywords:** Science Policy & Publishing

## Abstract

*EMBO Reports* was amongst the pioneers of the short scientific article: 25 years later, the format is needed more than ever.

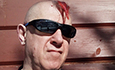

When I was invited to contribute this column in celebration of *EMBO Reports*’ first 25 years, I first toyed with the idea of writing retrospectively, from a seriously dystopian future, about the second 25 years of the journal. But, on reflection, I felt that to expand the literature further with an account of what may never come to pass, even if rooted in the worrying trends of today, just adds to the increasing burden of sifting truth from falsehood.

So I have decided to focus instead on something more positive. Something that I strove to implement during my years as Chief Editor, which I feel is still a live issue in scientific publication: namely, the virtues of short-format articles, which I espoused in one of my early editorials “Short is Sweet” (Jacobs, 2009).

In essence, I advocated the idea that the best kind of ground-breaking research communication presents a single key finding, backed up by multiple lines of experimental evidence. *EMBO Reports* was among the pioneers of the short-format article in the molecular life sciences. Some other publishing houses have experimented with the concept, although ‘XXXXX Reports’ often seems to mean something else. The original idea of a short-format article has gradually been replaced by an emphasis on scientific validity over “impact”. Despite nominal length restrictions, articles in many scientific journals seem unnecessarily long and often rather stodgy, with numerous “supplementary” figures that are nevertheless integral to the work being presented. The real impact of such works is muted by their expanded and convoluted format, regardless of their actual content.

Too often, reviewers—backed up by editors—insist on additional experiments, sometimes peripheral to the main point of a paper, that support their own pre-judgment rather than the one actually indicated by the data. The main finding eventually gets diluted or even obscured. While it is true that peer review almost invariably improves the quality of published manuscripts, this kind of diversion can make the literature hard to follow and detracts from real impact.

Manuscripts that are already lengthy and heterogeneous at an early stage are exhausting to read and evaluate, potentially compromising the quality of peer review. While I am not suggesting that authors and editors deliberately compile overly voluminous articles to confuse peer reviewers, the effect on the reader can be off-putting. If a finished scientific article is too bulky or meandering to read, we tend to rely too heavily on a short abstract, author summary or editorial blurb, rather than look critically at the actual contents.

On balance, I feel that a rethink is needed. At least in my current role as Editor-in-Chief of *Fly*, the benchmark journal in *Drosophila* biology, I have sought to encourage short-format submissions (Jacobs, 2020).

There is also an obvious danger in the opposing trend, towards shorter and shorter ‘papers’ that merely present the findings of a single experiment or even just an abbreviated compilation of data collected with no specific hypothesis or purpose in mind. The literature is growing at an alarming rate, and the proliferation of micro-publications of this type is making the problem worse. We are coming to rely increasingly on the use of AI tools that are still too crude and unmoderated to conduct a meaningful evaluation of the significance of such communications that might equate to proper peer review.

To address this problem, I have also considered the possibility that we could replace much of the published literature with compendia of career-long sets of inter-related findings. This is close to the original idea of a PhD thesis, except that it would be unlimited in size and time, a body of work that scientists could extend and amend periodically, that would complement short-format articles that report major conceptual advances along the way.

We are already comfortable with the idea that artists can paint over works with which they are not satisfied and may do so repeatedly. Some of the most famous paintings in the world are now known to be just the latest version of a series of imperfect precursors. So why not a body of experimental work on mtDNA replication, the alternative respiratory chain, or mitochondrial heat production? We could each have a few major compendium papers that can be continuously updated, eliminating conceptual wrong turnings, data that turned out to be spurious or artifactual, or superseded by more thorough or accurate data acquired through improved methods. And revised interpretations that fit the expanded body of experimental findings.

Some may argue that every successive amendment to such a compendium would be considered a “retraction”, and thus an implicit admission of incompetence or misconduct. But I beg to disagree. Science is not an immutable canon. We need to get away from the idea that it is. Deductions must evolve in the light of new results and more advanced methods. We should be proud, not embarrassed, to say “I changed my mind” or “How naive we were”. This is the very essence of science, which distinguishes it from religion. Of course, we should still identify mistakes and cases of outright fraud: that is the purpose of peer review and public scrutiny after publication. But to expect every published work to remain accurate and uncontestable 5, 20, or 50 years later is unrealistic and damaging to scientific progress.

Short is still sweet, but science can also be salty or spicy—though hopefully not sour. As *EMBO Reports* glides into its second quarter-century my hope is that it continues to experiment with new formats and concepts in publishing: including a renewed emphasis on short format, but with a conscious aim of satisfying increasingly overwhelmed readers, desperate to extract meaning from the rapidly growing scientific literature.
